# Validation of mid-infrared spectroscopy for continuous glucose monitoring: the manage II study

**DOI:** 10.1186/2197-425X-3-S1-A294

**Published:** 2015-10-01

**Authors:** J-C Preiser, A Brasseur, D Fagnoul, J-L Vincent

**Affiliations:** Intensive Care, Erasme University Hospital, Brussels, Belgium; Erasme University Hospital, Brussels, Belgium

## Introduction

Intravascular continuous glucose monitoring (CGM) in critically ill patients represents a major advance to improve the quality of care and to decrease the nursing workload dedicated to glycemic control. However, the presence of several physical or chemical interferences can limit the accuracy of the blood glucose readings (BG) measured by the CGM, implying the need for a thorough validation process for each technique or device used for CGM.

## Objectives

To assess the accuracy of Optiscanner (Optiscan Corp, Hayward, CA), which directly measures mid infrared absorption of glucose in plasma.

## Methods

Adult medical and surgical critically ill patients with an expected length of stay in the ICU of at least 3 days, an admission BG > 150 mg/dl and a need for central venous and arterial catheterization were enrolled after signed informed consent. A 4-lumen central venous catheter was inserted into the superior vena cava and the proximal port was connected to the OptiScanner. Blood was drawn every 15 minutes and BG was determined on 0.1 mL of centrifuged blood by mid-infrared spectroscopy. Comparative samples were drawn 12 times per day separated by a minimum 60-min time interval. BG reference values were measured by two different blood gas analysers (YSI 2300 STAT Plus and GEM4000). BG measured with the Optiscanner were plotted against the reference BG on a Clarke error grid (CEG). The percentage of values in the A and B zones of the CEG, i.e when BG values were within 20% of the reference sensor, or when the inaccuracy would not influence the treatment, and the mean amplitude of the relative difference (MARD) were determined.

## Results

Eighty-five patients (median age 63 years (range 21-88), predominantly males (65%) with a median APACHE II score of 20 (range 6-41) were included. The ICU and hospital mortality were 19 and 20 %, respectively. 8,430 BG values were determined by the OptiScanner and 862 paired BG, ranging from 60 to 352 mg/dl, values were analysed. The percentage of values between 70 and 150 mg/dl were 60% and 58.6% when measured by the OptiScanner and the blood gas analyzers, respectively. The percentage of values in the A and B zones of the CEG were 95.1 and 4.8% The MARD was 8%.

## Conclusions

The Optiscanner provided reliable and accurate BG values over a large range of values in a heterogeneous population of ICU patients. The impact of a therapeutic strategy using this device on glucose control performance and indices of nursing workload should be studied.

